# Guideline-recommended basic parameter adherence in neurocritical care stroke patients: Observational multicenter individual participant data analysis

**DOI:** 10.1177/23969873241289360

**Published:** 2024-10-13

**Authors:** Anne Mrochen, Omar Alhaj Omar, Johann O Pelz, Dominik Michalski, Hermann Neugebauer, Dominik Lehrieder, Benjamin Knier, Corinna Ringmaier, Henning Stetefeld, Silvia Schönenberger, Min Chen, Hauke Schneider, Angelika Alonso, Hendrik Lesch, Andreas Totzek, Friedrich Erdlenbruch, Benedikt Hiller, Norma J Diel, André Worm, Christian Claudi, Stefan T Gerner, Hagen B Huttner, Patrick Schramm

**Affiliations:** 1 Department of Neurology, Justus-Liebig-University, Giessen, Germany; 2 Translational Neuroscience Network Giessen (TNNG), Giessen, Germany; 3 Department of Neurology, University Hospital of Leipzig, Leipzig, Germany; 4 Department of Neurology, University of Würzburg, Würzburg, Germany; 5 Department of Neurology, Technical University of Munich School of Medicine, Munich, Germany; 6 Department of Neurology, Faculty of Medicine and University Hospital Cologne, University of Cologne, Cologne, Germany; 7 Department of Neurology, Heidelberg University Hospital, Heidelberg, Germany; 8 Department of Neurology, University Hospital Augsburg, Augsburg, Germany; 9 Medical Faculty, University of Technology, Dresden, Germany; 10 Department of Neurology, University Hospital Mannheim, University of Heidelberg, Mannheim, Germany; 11 Department of Neurology, Center for Translational Neuro- and Behavioral Sciences (C-TNBS), University Hospital Essen, University Duisburg-Essen, Essen, Germany; 12 Center of Mind, Brain and Behavior (CMBB), Marburg, Germany

**Keywords:** NICU, bundle, adherence, ICH, AIS

## Abstract

**Introduction:**

Neurocritical care patients with neurovascular disease often face poor long-term outcomes, highlighting the pivotal role of evidence-based interventions. Although International Guidelines emphasize managing basic physiological parameters like temperature, blood glucose, blood pressure, and oxygen levels, physician adherence to these targets remains uncertain. This study aimed to assess adherence to guideline-based treatment targets for basic physiological parameters in neurocritical care.

**Patients and Methods:**

This multicenter observational study was conducted across eight tertiary University Hospitals in Germany analyzed 474 patients requiring mechanical ventilation (between January 1st and December 31st, 2021). Adherence was defined as the rate of measurements within therapeutic ranges for systolic blood pressure (situation-adapted), mean blood pressure (MAP, 60–90 mmHg), glucose levels (80–180 mg/dl), body temperature (<37.5°C), partial arterial pressure of oxygen (PaO_2_) 80–120 mmHg und partial arterial pressure of carbon dioxide (PaCO_2_) 35–45 mmHg during the initial 96 h of hospitalization in 4 hour-intervals.

**Results:**

Overall, 70.7% of all measurements were within the predetermined therapeutic ranges including SBP (71.3%), temperature (68.3%), MAP (71.4%), PaO_2_ (65.2%), PaCO_2_ (75.0%) and blood glucose (80.7%).

**Discussion and Conclusion:**

This multicenter study demonstrates adherence to guideline-based treatment targets, underscoring the high standards maintained by neurological intensive care units. Our study offers valuable insights into adherence to guideline-based treatment targets for neurocritical care patients in Germany. To improve patient care and optimize therapeutic strategies in neurovascular diseases, further research is needed to examine the impact of these adherence parameters on long-term outcomes.

## Graphical abstract

**Graphical abstract:**
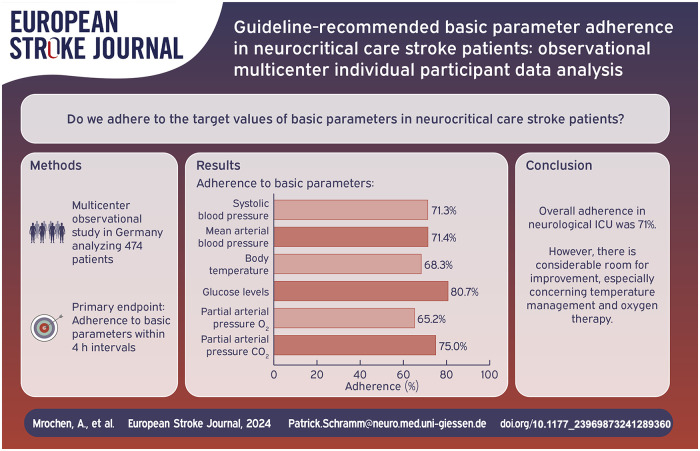

## Introduction

Neurovascular disease patients in intensive care often face poor long-term outcomes due to factors like advanced age, comorbidities, and irreversible neuronal loss.^1,2^ Given the scarcity of evidence-based treatments, it is crucial to prioritize interventions with strong evidence, such as decompressive surgery for acute ischemic stroke, reversal of anticoagulation in intracerebral hemorrhage, and early aneurysm closure in subarachnoid hemorrhage.^3–6^ However, regardless of the disease type, managing “basic physiological parameters” such as temperature, glucose, blood pressure, and oxygen levels is fundamental in neurocritical care unit (NICU) treatment. Yet, evidence supporting adherence to recommended guidelines concerning long-term functional neurological outcomes in neurovascular neurocritical care patients is still lacking.^7–9^

Furthermore, there is currently growing interest in treatment bundles as evidence suggests that individual parameters alone may not suffice to significantly impact outcomes.^10^ As currently demonstrated, the implementation of standard operating procedures (SOPs) to adhere to these basic parameters has been shown to improve outcomes for patients with intracerebral hemorrhage in low-income countries.^10^ Yet, it is still uncertain (i) how stringently the recommended target values of basic parameters are adhered to in daily routine management and (ii) which factors are related to adherence.

Here, we performed a multicenter observational individual participant data analysis of patients with acute ischemic stroke (AIS), intracerebral hemorrhage (ICH) and subarachnoid hemorrhage (SAH) treated at dedicated neurocritical care units in Germany. We aimed to (i) analyze the adherence to disease-specific guideline-recommendations for management of “basic NICU parameters” including systolic blood pressure (SBP), mean arterial blood pressure (MAP), temperature, blood glucose and blood oxygenation, including partial arterial pressure of oxygen (PaO_2_) and partial arterial pressure of carbon dioxide (PaCO_2_) and (ii) to evaluate factors associated with parameter-adherence.

## Methods

### Study design and population

We performed a retrospective observational multicenter study of neurointensive care patients requiring mechanical ventilation treated at eight dedicated neurocritical care units of tertiary University Hospitals in Germany. The study was approved by the institutional review boards and local ethics committees of all participating sites based on the central vote from Giessen University (AZ 177122). Data collection covered the period from 1st January until 31th December, 2021. Patients with the following inclusion criteria were enrolled: (i) age > 18 years; (ii) acute neurovascular disease, i.e. cerebral ischemia, intracerebral hemorrhage or subarachnoid hemorrhage (International Classification of diseases version 10, ICD10, i.e., 160.x, 161.x, I62.x, 163.x), (iii) neurocritical care admission due to intubation and controlled ventilation, and (iv) a hospital stay on NICU of a minimum of 4 days. Patients who received initial do-not-treat/do-not-resuscitate (DNT/DNR) orders as well as those who deceased within 24 h after admission were not enrolled. A priori we excluded all patients with missing data documentation (>33% missing data) or availability of the 4-hour measures during the first 96 h of treatment.

### Clinical parameters

All obtained clinical parameters were extracted from institutional databases. These parameters were determined upon admission and every 4 h for the first 96 h of intensive care treatment. We assessed baseline demographic parameters (age, gender) as well as type of neurovascular disease including clinical parameters upon admission and pre-existing disability (measured using the modified Rankin Scale, mRS).^11^ Specifically, we focused on the following parameters measures within 4 h intervals: (i) blood pressure, including systolic (SBP) and mean arterial blood pressure (MAP), (ii) temperature as obtained via bladder catheters, (iii) blood glucose, and (iv) blood oxygenation, including partial arterial pressure of oxygen (PaO_2_) and partial arterial pressure of carbon dioxide (PaCO_2_). Associated parameters consisted of duration of ventilation, duration of hospital stay, National Institute of Health Stroke Scale (NIHSS) score at discharge and in-hospital mortality and thrombolysis in cerebral infarction scale (TICI).^12–15^ Patient parameters were anonymized upon entry into the pooled individual participant data sheet.

### Outcomes

The primary endpoint was adherence to the guideline-based treatment-target recommendations, measured as the rate of parameters within recommended ranges divided by all measurements of the respective parameter, i.e. as percentage. Specifically, for patients with cerebral ischemia, we adhered to the guidelines established by the German Society of Neurology, targeting a systolic blood pressure (SBP) range of 120–180 mmHg.^16^ Following the recommendations of the European Stroke Organization (ESO) for acute ischemic stroke management, after successful thrombectomy (TICI 3), our SBP target range was adjusted to 110–160 mmHg.^17^ In cases where acute reperfusion treatment was not implemented or was unsuccessful, SBP values exceeding 180 mmHg within the first 24 h were deemed acceptable. Additionally, the initial SBP measurement post-admission was considered “non-adherent” if it exhibited a reduction of >25% compared to the admission SBP.^17^ Furthermore, our protocol included maintaining mean arterial pressure (MAP) between 60 and 90 mmHg, controlling temperature (<37.5°C), managing blood glucose levels (80–180 mg/dl), and ensuring arterial oxygen partial pressure (PaO_2_) between 80 and 120 mmHg and carbon dioxide partial pressure (PaCO_2_) between 35 and 45 mmHg.^16,18^ For hemorrhagic stroke patients, our targeted parameters are: SBP (ICH: 110–140 mmHg; SAH: 110–180 mmHg), MAP (60–90 mmHg), temperature (<37.5°C), blood glucose (80–180 mg/dl) SAB,^7,10,17,19–21^ as well as blood gases PaO_2_ (80–120 mmHg) and PaCO_2_ (35–45 mmHg) according to the recommendations of the European Society of Intensive Care Medicine.^18^ Additionally, the initial SBP measurement post-admission was considered “non-adherent” if it exhibited a reduction of >90 mmHg compared to the admission SBP.^17^

### Statistics

All statistical analyses were conducted using the SPSS software package version 29 (www.spss.com). The level of significance was set at alpha = 0.05. Continuously monitored data, including SBP measured via arterial lines, MAP, and temperature were recorded as discrete values at least once within each respective 4-hour interval. Discontinuously collected data, such as blood gas analyses and blood glucose measurements, were assigned to the nearest corresponding 4-hour time point. Data were presented as median and interquartile ranges as well as total counts with percentage. Adherence was measured as the rate of parameters within recommended ranges divided by all measurements of the respective parameter, that is, as percentage. To investigate patient characteristics associated with parameter adherence, we used multivariate regression analysis, adjusting for NIHSS, age, and sex with adherence as a median split binary variable.^22,23^ To compare the respective center-specific adherence rates, we used a one-way ANOVA.

## Results

### Study population

**Figure 1.**
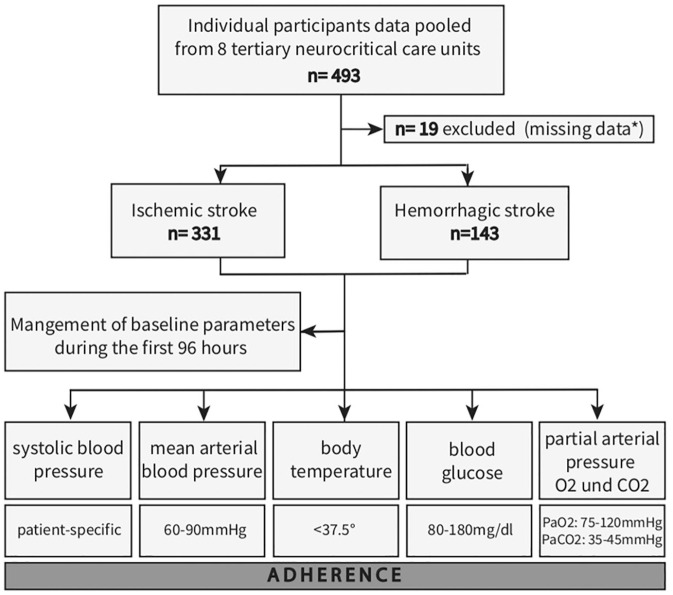
Flow chart of study participants. A total of 493 neurocritical care patients from eight tertiary University Hospitals in Germany were investigated during the period from January 1st to December 31st, 2021. Nineteen patients were excluded because of missing data. Among the 474 patients remaining, 331 were diagnosed with ischemic stroke, while 143 had hemorrhagic stroke. Clinical parameters, including systolic blood pressure, mean arterial blood pressure, body temperature, blood glucose levels, and partial arterial pressure of oxygen and carbon dioxide, were recorded upon admission and every 4 h for the initial 96 h of intensive care treatment. *Missing data consisted of: missing data documentation (>33% missing data) or availability of the 4-hour measures during the first 96 h of treatment.

**Figure 2.**
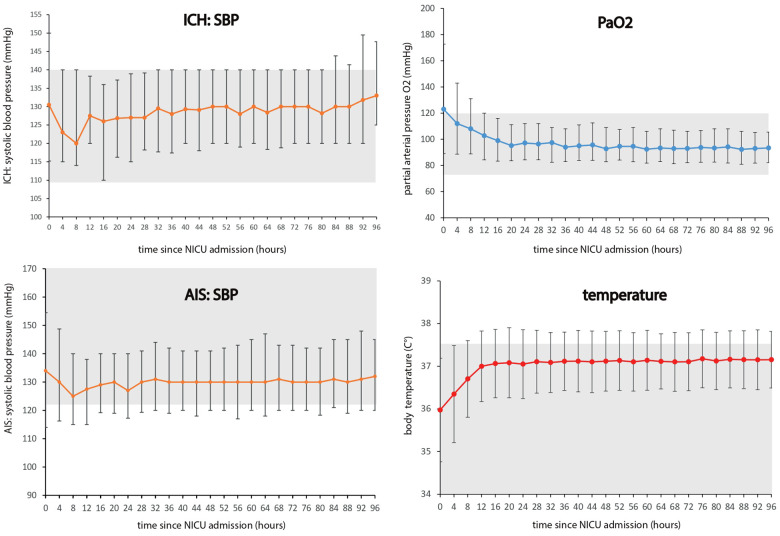
Median values of basic clinical parameters in 4-hours intervals during the first 96 h. AIS: acute ischemic stroke; ICH: intracerebral hemorrhage; PaO_2_: partial arterial pressure of oxygen; SBP: systolic blood pressure; NICU: neurointensive care unit. Median values (IQR) for systolic blood pressure, mmHg separated for intracerebral hemorrhage and acute ischemic stroke, partial pressure of oxygen, mmHg and temperature, °C measured in 4-hours intervals since admission during the first 96 h.

Between January 1, 2021, and December 31, 2021, a comprehensive analysis was conducted on a total of 474 patients diagnosed with ischemic and hemorrhagic stroke across eight participating centers (Figure 1). Demographic and clinical characteristics of the study population are summarized in Table 1. The mean age of the cohort was 68.3 (SD 13.8) years, with 42.2% (200/474) being female. Upon admission, patients presented with a median NIHSS score of 19, and a significant portion (69.6% (330/474)) required preclinical intubation. Ischemic stroke was the predominant diagnosis, accounting for 69.8% (331/474) of cases, while hemorrhagic stroke constituted 30.2% (143/474), with intracerebral hemorrhage being the most prevalent subtype (24.1% or 114/474). In terms of interventions, intravenous thrombolysis was performed in 30.2% (100/331) of ischemic stroke cases, while 67.1% (223/331) underwent endovascular therapy. Among hemorrhagic stroke patients, external ventricular drains were utilized in 63.6% (91/143) of cases, and surgical hematoma evacuation was performed in 23.1% (33/143). Regarding outcomes, the median NIHSS score at discharge was 18. The in-hospital-mortality rate was 42.4% (201/474). All patients included in this study were mechanically ventilated during the observation period of 96 h. The median duration of ventilation was 235 h (IQR 137–378). We graphically displayed initial and subsequent measurements (median, interquartile range) over 96 h for SBP, MAP, temperature, blood glucose, PaO_2_ and PaCO_2_ (Figure 2, Supplemental Figure 1) within 4-hours intervals. With exception of initial median arterial PaO_2_ (PaO_2_ value, 123 mmHg (IQR, 89–173 mmHg)) the median values for all other parameters fall within their respective predefined target range (shaded in grey, Figure 2, Supplemental Figure 1). The interquartile range reveal variability, with outliers in ischemic stroke mostly falling within the lower blood pressure range and in hemorrhagic stroke in the upper range. For temperature, outliers are mainly in the upper range. In arterial blood gas measurements, PaCO_2_ outliers appear both above and below the target range, with initial PaO_2_ measurements trending towards hyperoxemia.

**Table 1.** Baseline characteristics and outcome parameters of the overall cohort.

	Cohort (*n* = 474)
*Baseline characteristics*	
Age,^a^ years	68.3 (13.8)
Sex,^b^ female	200 (42.2)
*Admission status*	
NIHSS at admission (0–42)^c^	19 (12–30)
Pre-mRS^c^	0 (0–2)
Prehospital intubation^b^	330 (69.6)
*Diagnosis*	
Ischemic stroke^b^	331 (69.8)
Hemorrhagic stroke^b^	143 (30.2)
ICH^b^	114 (24.1)
SAH^b^	29 (6.1)
*Disease specific interventions*	
EVT (Ischemic stroke)^b^	223/331 (67.1)
IVT (Ischemic stroke)^b^	100/331 (30.2)
Decompressive craniectomy (Ischemic stroke)^b^	11/331 (3.2)
Surgical hematoma evacuation (Hemorrhagic stroke)^b^	33/143 (23.1)
Coiling (Hemorrhagic stroke)^b^	22/143 (15.4)
Clipping (Hemorrhagic stroke)^b^	6/143 (3.5)
EVD (Hemorrhagic stroke)^b^	91/143 (63.6)
Lumbar drain (Hemorrhagic stroke)^b^	30/143 (21.0)
*Outcome parameter*	
NIHSS at discharge^c^	18 (9–25)
In-hospital mortality^b^	201 (42.4)
Duration of ventilation (h)^c^	234.5 (136.5–378)

EVD: external ventricular drain; EVT: endovascular therapy; IVT: intravenous thrombolysis; ICH: intracerebral haemorrhage; IQR: interquartile range; mRS: modified Rankin scale (0 no deficit to 6 death); NIHSS: National Institutes of Health Stroke Scale (ranging from 0, no deficit, –40, severe neurological deficit; 40 is the maximum because in comatose ataxia is not scored), applied for patients with ischemisch stroke and intracerebral haemorrhage; SAH: subarachnoidal haemorrhage.

a Mean ± SD.

b *n* (%).

c Median (interquartile range: 25th–75th percentile).

### Adherence patterns to guideline-based treatment targets

We assessed adherence to guideline-based treatment-target recommendations within 4-hour intervals for key physiological parameters. Overall, 70.7% of all measurements were within the predetermined therapeutic ranges. Our findings indicate that out of 474 patients, 1 (0.2%) had adherence levels between 30% and 39%, 12 (2.5%) between 40% and 49%, 46 (9.7%) between 50% and 59%, 132 (27.8%) between 60% and 69%, 217 (45.8%) between 70% and 79%, 64 (13.5%) between 80% and 89%, and 2 (0.4%) had adherence levels of 90% and 99%.

**Figure 3.**
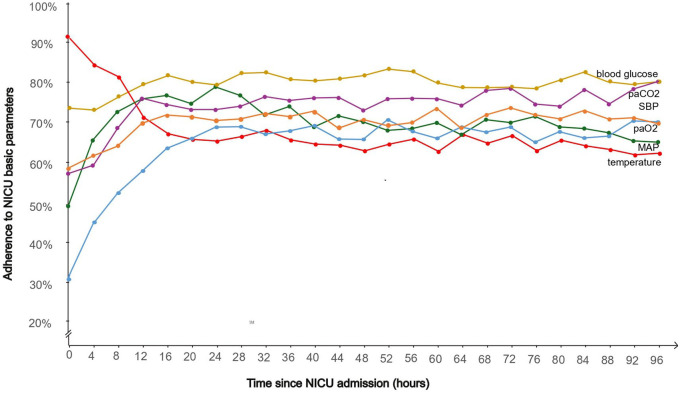
Adherence to guideline-based treatment targets in 4-hours intervals during the first 96 h of NICU. SBP: systolic blood pressure; MAP: mean arterial pressure; PaO_2_: partial arterial pressure of oxygen; PaCO_2_: partial arterial pressure of carbon dioxide; NICU: neurointensive care unit. Adherence to guideline-based treatment targets, that is, SPB, MAP, temperature, blood glucose, PaCO_2_ and PaO_2_ in 4-hours intervals during the first 96 h of neurocritical care treatment. Adherence is determined as the percentage (%) of measurements within the guideline-recommended range relative to the total measurements assessed within 4-hours intervals.

Table 2 and Figure 3 provide a comprehensive daily and 4 hour-interval analysis of adherence rates to guideline-based treatment targets over the initial 96 h of intensive care treatment. Our findings showed dynamic temporal adherence patterns across different parameters, with fluctuations observed during the first four days of treatment.

**Table 2.** Adherence of NICU parameters from admission to day 4.

	Systolic blood pressure (%)	Mean arterial blood pressure (%)	Body temperature (%)	Glucose levels (%)	Partial arterial pressure O_2_ (%)	Partial arterial pressure CO_2_ (%)
Admission	58.7	50.6	91.6	74.4	30.6	58.2
Day 1	69.4	75.5	73.2	79.2	59.6	75.7
Day 2	72.6	73.7	66.0	82.2	68.7	76.1
Day 3	72.6	70.5	66.0	81.2	69.6	77.3
Day 4	72.8	69.3	64.0	81.0	68.9	77.5
Overall	71.3	71.4	68.3	80.7	65.2	75.0

Adherence to basic parameters is presented daily and cumulatively over a 96-hour period. Adherence is determined as the percentage (%) of measurements within the guideline-recommended range relative to the total measurements assessed upon admission, on a daily basis and overall.

Adherence to SBP, MAP, blood glucose levels, PaO_2_, and PaCO_2_ generally improved from admission to the first day, indicating effective initial adjustments in treatment strategies. In contrast, adherence to temperature was notably high upon admission at 91.6%, but declined to 73.2% by day 1 and further to 64.0% by day 4. Overall, blood glucose levels consistently exhibited the highest adherence rates, followed by PaCO_2_ and SBP. Initial adherence to PaO_2_ was low, primarily due to hyperoxygenation with levels exceeding 120 mmHg.

### Adherence associated parameters

Factors associated with high adherence to guideline-based treatment targets for physiological parameters in neurocritical care were comprehensively investigated using multivariate regression analysis. Among AIS and AHS we found no association regarding age, sex, NIHSS, pre-mRS, stroke subtype or TICI. The center-specific adherence rates ranged between 62.8% and 78.1% with significant difference between centers (*F* = 15.49, *p* < 0.05), see Supplemental Figure 2.

## Discussion

This study represents the first comprehensive investigation of adherence to guideline-based treatment targets for neurocritical care patients with neurovascular disease in Germany, encompassing a broad representation of tertiary care centers. Our key findings reveal: (i) neurological intensive care units maintain high standards, yet there is room for improvement; and (ii) adhering to strict temperature targets remains a significant challenge. While all other parameters rather improved in regard to their respective recommended thresholds during course of disease, temperature was the only parameter with decreasing adherence over time. Two aspects emerge from the data.

First, the adherence to certain thresholds in neurointensive care has been recommended for decades.^24–31^ While this was initially based on pathophysiological considerations, several subsequent studies have provided varying levels of robust evidence supporting the recommended guidelines.^32–34^ Nonetheless, the rationale for adhering to these recommended thresholds was also based on non-clinical outcomes, such as surrogate measures like cerebrovascular autoregulation, intracranial pressure, edema formation, and similar parameters.^35–39^ Although, strictly speaking, evidence for adhering to the recommended guidelines with respect to long-term functional neurological outcome of neurovascular neurocritical care patients is still lacking, it is however highly likely that patients should benefit if adherence levels are rather high. Hence, our findings add knowledge to the field, as we here provide a first comprehensive analysis of the current situation across dedicated neurointensive care units in Germany. In essence, adherence levels appear acceptable, but need improvement. The latter seem achievable through interventions such as the establishment and implementation of straightforward Standard Operating Procedures (SOPs). As demonstrated in prior studies,^40^ these might help further improve and increase average adherence levels. Furthermore, it should be noted that the evidence levels for different parameters vary, which should be considered when interpreting adherence in this study. While evidence on blood pressure management within the initial 24 h is robust, particularly for patients receiving reperfusion therapy for acute ischemic stroke and those with intracerebral hemorrhage (ICH), evidence for other parameters like temperature management or oxygenation targets varies and is often weak.^17,41^ Notably, the INTERACT 3 trial serves as the pioneering randomized controlled trial (RCT) in patients with intracerebral hemorrhage (ICH) to unveil the advantages of a “bundle care” intervention.^10^ However, it is noteworthy that this trial was conducted in low- and middle-income countries, thereby potentially limiting its applicability to high-income countries where adherence to standardized treatment protocols is more prevalent. The subsequent INTERACT 4 trial further contributes to this field by investigating the effects of prehospital blood-pressure reduction, which did not improve functional outcomes in a cohort of patients with undifferentiated acute stroke.^42^ Our analysis of center-specific effects at established tertiary care centers in Germany, with adherence rates ranging from 62.8% to 78.1%, reveals certain differences in daily routine, most likely because of diverging SOPs. Hence, establishing strict protocols with teaching of the nursing and physician teams regarding monitor alarms and seem to indeed hold promise in enhancing overall adherence. Nevertheless, the value of adherence necessitates further enhancement and assessment in terms of its impact on relevant long-term functional clinical endpoints.

Second, interestingly variations in adherence were observed across different parameters. Parameters such as blood glucose and PaCO_2_ levels demonstrated acceptable adherence, while blood pressure (both SBP and MAP) showed improvement over time but still require further enhancement. Temperature management emerged as a critical concern, as the initial measurement indicated mild hypothermia, yet adherence to this target declined which has been shown to have adverse effects.^41,43–46^ The strong correlation between brain damage and fever increases notably within the first 24 h, potentially contributing to lower adherence observed, indicative that management may be inadequate when fever occurs.^43^ One possible reason for the low level of adherence rates could be hesitancy due to the relatively high costs of devices, along with the need for deeper sedation to achieve the target of normothermia. Additionally, the lack of robust efficacy data, particularly in the context of ischemic stroke, may also contribute to the low adherence rates.

Furthermore, initial oxygenation performance was found to be the poorest, with only 30.6% adherence at admission. However, this is primarily due to excessive oxygen administration, which is known to be detrimental due to the production of free radicals, among other factors.^47^ Therefore, simply aiming for oxygen levels above 80 mmHg is insufficient, as it often leads to excessively high values. Addressing this issue requires interventions to prevent hyperoxia and optimize oxygen therapy strategies.

While these data were collected from multiple centers, a limitation of the study is the small sample size for exploratory analyses and the inherent limitations associated with the retrospective nature of this cohort study. Additionally, the study design did not allow for a time-based assessment of adherence. Ideally, adherence would be defined as making a correction within a specific time interval after detecting a pathological value, an approach that should be addressed in future prospective studies. Furthermore, dosages and frequencies of therapeutic medications were not included in the investigations. Bias due to confounding cannot be fully ruled out. Another limitation is the lack of time-related data, such as time from symptom onset to admission, real-time variability of parameters and short-term drops of blood pressure or partial arterial oxygen pressure which was not recorded.^48^ Additionally, critical aspects for preventing secondary brain injury, such as intracranial pressure monitoring, optimal timing for extubation decisions, and management strategies tailored to specific stroke subtypes, are not addressed in this study. While we focused on patient-specific characteristics that can influence adherence, there are many other factors, such as implementation strategies, clinical guidelines, and the broader environmental context, which future studies should explore in greater detail through prospective research. Furthermore, the study did not examine the impact of adherence on potential clinical outcomes, which should be addressed in future intervention studies, particularly in context of individualized therapy tailored on stroke subtype, etiology, and other relevant factors.^49^ However, despite these limitations, the study effectively addresses the straightforward question of adherence, fulfilling the intention of the study.

In conclusion, our study provides valuable insights into adherence to guideline-based treatment targets for neurocritical care patients in Germany. Variations in adherence across parameters underscore the need for tailored interventions, particularly in temperature management and oxygen therapy strategies.

## Supplemental Material

sj-docx-1-eso-10.1177_23969873241289360 – Supplemental material for Guideline-recommended basic parameter adherence in neurocritical care stroke patients: Observational multicenter individual participant data analysis Supplemental material, sj-docx-1-eso-10.1177_23969873241289360 for Guideline-recommended basic parameter adherence in neurocritical care stroke patients: Observational multicenter individual participant data analysis by Anne Mrochen, Omar Alhaj Omar, Johann O Pelz, Dominik Michalski, Hermann Neugebauer, Dominik Lehrieder, Benjamin Knier, Corinna Ringmaier, Henning Stetefeld, Silvia Schönenberger, Min Chen, Hauke Schneider, Angelika Alonso, Hendrik Lesch, Andreas Totzek, Friedrich Erdlenbruch, Benedikt Hiller, Norma J Diel, André Worm, Christian Claudi, Stefan T Gerner, Hagen B Huttner and Patrick Schramm in European Stroke Journal

sj-jpg-2-eso-10.1177_23969873241289360 – Supplemental material for Guideline-recommended basic parameter adherence in neurocritical care stroke patients: Observational multicenter individual participant data analysis Supplemental material, sj-jpg-2-eso-10.1177_23969873241289360 for Guideline-recommended basic parameter adherence in neurocritical care stroke patients: Observational multicenter individual participant data analysis by Anne Mrochen, Omar Alhaj Omar, Johann O Pelz, Dominik Michalski, Hermann Neugebauer, Dominik Lehrieder, Benjamin Knier, Corinna Ringmaier, Henning Stetefeld, Silvia Schönenberger, Min Chen, Hauke Schneider, Angelika Alonso, Hendrik Lesch, Andreas Totzek, Friedrich Erdlenbruch, Benedikt Hiller, Norma J Diel, André Worm, Christian Claudi, Stefan T Gerner, Hagen B Huttner and Patrick Schramm in European Stroke Journal

sj-jpg-3-eso-10.1177_23969873241289360 – Supplemental material for Guideline-recommended basic parameter adherence in neurocritical care stroke patients: Observational multicenter individual participant data analysis Supplemental material, sj-jpg-3-eso-10.1177_23969873241289360 for Guideline-recommended basic parameter adherence in neurocritical care stroke patients: Observational multicenter individual participant data analysis by Anne Mrochen, Omar Alhaj Omar, Johann O Pelz, Dominik Michalski, Hermann Neugebauer, Dominik Lehrieder, Benjamin Knier, Corinna Ringmaier, Henning Stetefeld, Silvia Schönenberger, Min Chen, Hauke Schneider, Angelika Alonso, Hendrik Lesch, Andreas Totzek, Friedrich Erdlenbruch, Benedikt Hiller, Norma J Diel, André Worm, Christian Claudi, Stefan T Gerner, Hagen B Huttner and Patrick Schramm in European Stroke Journal
