# Supplementary material for: Guideline-recommended basic parameter adherence in neurocritical care stroke patients: Observational multicenter individual participant data analysis
Source: Eur Stroke J. 2024 Oct 13:23969873241289360. Online ahead of print. doi: 10.1177/23969873241289360 (PMC11556612; doi:10.1177/23969873241289360)
Supplement: sj-docx-1-eso-10.1177_23969873241289360 – Supplemental material for Guideline-recommended basic parameter adherence in neurocritical care stroke patients: Observational multicenter individual participant data analysis [file sj-docx-1-eso-10.1177_23969873241289360.docx]

**Supplemental Figure 1. Median values of basic clinical parameters in 4-hours intervals during the first 96h.**

Median values (IQR) for mean arterial pressure, mmHg, partial pressure of carbon dioxide, mmHg and glucose levels, mg/dl measured in 4-hours intervals since admission during the first 96 hours.

Abbreviations: SBP; systolic blood pressure, MAP; mean arterial pressure, PaO2; partial arterial pressure of oxygen, PaCO2; partial arterial pressure of carbon dioxide, NICU; neurointensive care unit.

**Supplemental Figure 2. Center-specific Adherence of NICU parameters.**

Center-specific (Center 1- 8) overall adherence to guideline-based treatment targets, i.e. SPB, MAP, temperature, blood glucose, PaCO2 and PaO2 in 4-hours intervals during the first 96 hours of neurocritical care treatment. Adherence is determined as the percentage (%).

Abbreviations: SBP; systolic blood pressure, MAP; mean arterial pressure, PaO2; partial arterial pressure of oxygen, PaCO2; partial arterial pressure of carbon dioxide, NICU; neurointensive care unit.
