# Supplementary figures and images for: Guideline-recommended basic parameter adherence in neurocritical care stroke patients: Observational multicenter individual participant data analysis
Source: Eur Stroke J. 2024 Oct 13:23969873241289360. Online ahead of print. doi: 10.1177/23969873241289360 (PMC11556612; doi:10.1177/23969873241289360)

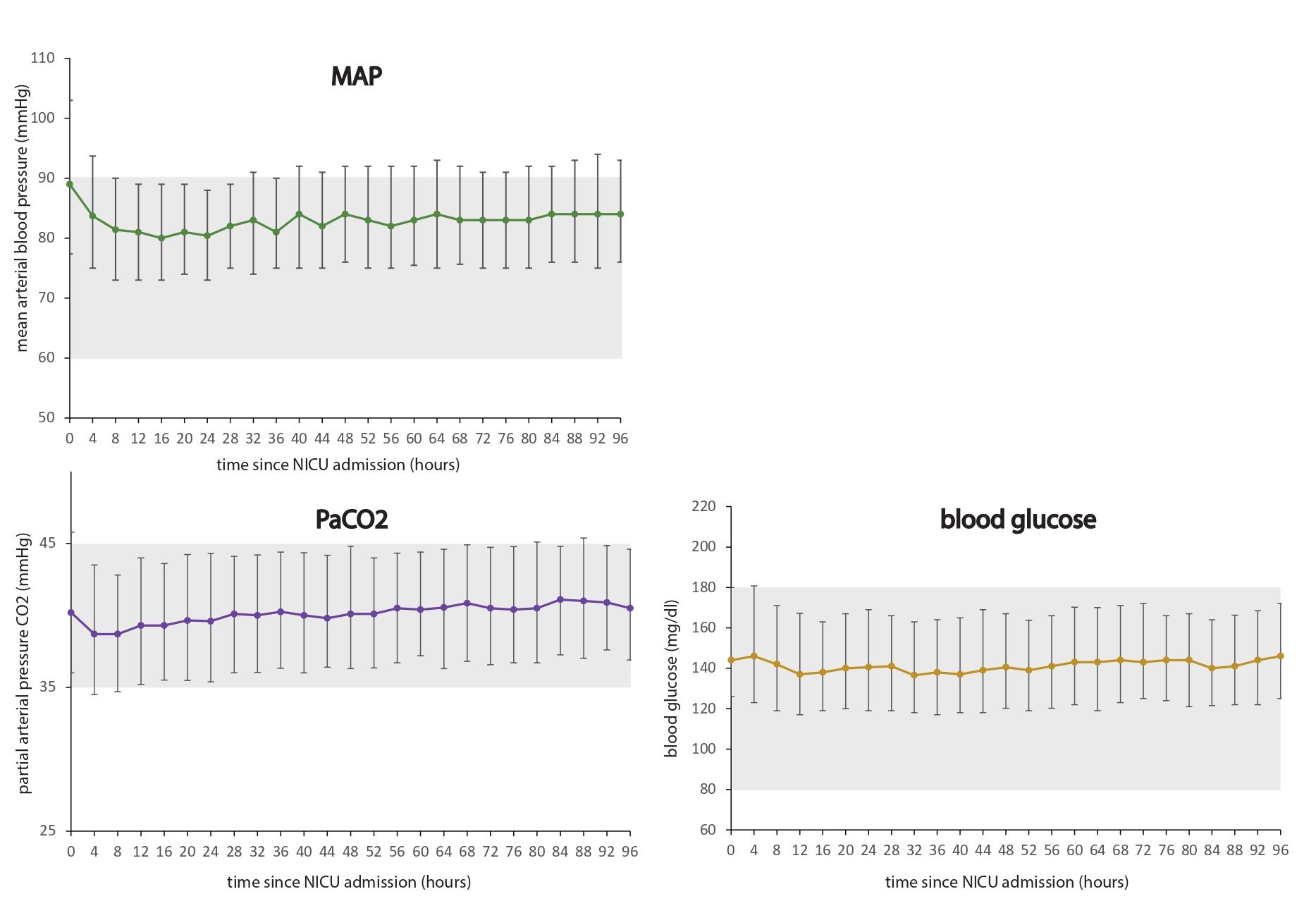

Supplement: sj-jpg-2-eso-10.1177_23969873241289360 – Supplemental material for Guideline-recommended basic parameter adherence in neurocritical care stroke patients: Observational multicenter individual participant data analysis [file sj-jpg-2-eso-10.1177_23969873241289360.jpg]

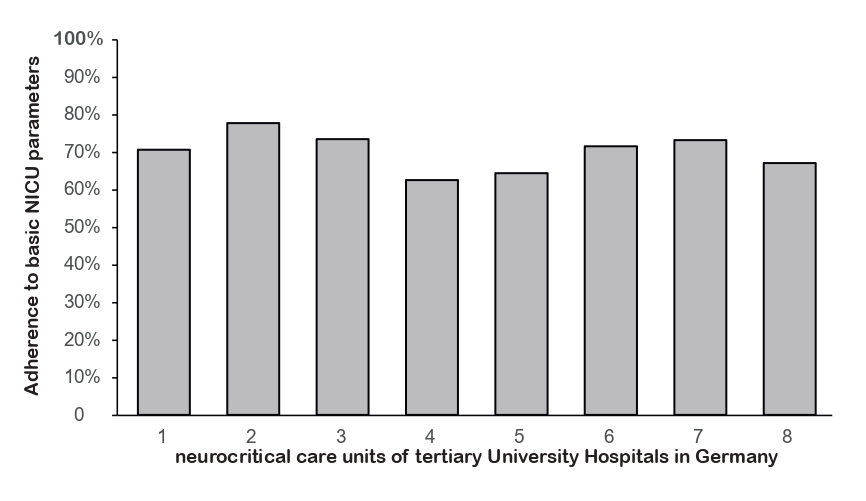

Supplement: sj-jpg-3-eso-10.1177_23969873241289360 – Supplemental material for Guideline-recommended basic parameter adherence in neurocritical care stroke patients: Observational multicenter individual participant data analysis [file sj-jpg-3-eso-10.1177_23969873241289360.jpg]
